# Supplementary material for: Modeling glioblastoma heterogeneity as a dynamic network of cell states
Source: Mol Syst Biol. 2021 Sep 16;17(9):e10105. doi: 10.15252/msb.202010105 (PMC8444284; doi:10.15252/msb.202010105)
Supplement: Supplementary file 5 — Source Data for Figure 3 [file MSB-17-e10105-s001.zip › Figure3A_sourcedata/GSEA_3065/nosigdb_state6.GseaPreranked.1622718443993/RICHARDS_NOWAKOWSKI_SCIENCE_2017_NIN1_UPREG.html]

Details for gene set RICHARDS\_NOWAKOWSKI\_SCIENCE\_2017\_NIN1\_UPREG[GSEA]

|  || Dataset | state6 |
| Phenotype | NoPhenotypeAvailable |
| Upregulated in class | na\_pos |
| GeneSet | RICHARDS\_NOWAKOWSKI\_SCIENCE\_2017\_NIN1\_UPREG |
| Enrichment Score (ES) | 0.34290183 |
| Normalized Enrichment Score (NES) | 1.5381079 |
| Nominal p-value | 0.0 |
| FDR q-value | 0.03710927 |
| FWER p-Value | 0.579 |
Table: GSEA Results Summary

  

Fig 1: Enrichment plot: RICHARDS\_NOWAKOWSKI\_SCIENCE\_2017\_NIN1\_UPREG      
 Profile of the Running ES Score & Positions of GeneSet Members on the Rank Ordered List

  

| PROBE | GENE SYMBOL | GENE\_TITLE | RANK IN GENE LIST | RANK METRIC SCORE | RUNNING ES | CORE ENRICHMENT || 1 | SOX4 |  |  | 3 | 0.375 | 0.0318 | Yes |
| 2 | BTG1 |  |  | 4 | 0.348 | 0.0615 | Yes |
| 3 | RPL30 |  |  | 34 | 0.260 | 0.0804 | Yes |
| 4 | RPL34 |  |  | 41 | 0.251 | 0.1012 | Yes |
| 5 | SOX11 |  |  | 70 | 0.230 | 0.1177 | Yes |
| 6 | RPS3A |  |  | 80 | 0.224 | 0.1358 | Yes |
| 7 | GLCCI1 |  |  | 101 | 0.213 | 0.1517 | Yes |
| 8 | HNRNPA1 |  |  | 108 | 0.209 | 0.1689 | Yes |
| 9 | TET2 |  |  | 150 | 0.192 | 0.1806 | Yes |
| 10 | POLR2J |  |  | 190 | 0.180 | 0.1914 | Yes |
| 11 | GLUL |  |  | 199 | 0.177 | 0.2056 | Yes |
| 12 | PARP1 |  |  | 206 | 0.175 | 0.2199 | Yes |
| 13 | RPL23A |  |  | 246 | 0.163 | 0.2293 | Yes |
| 14 | ELAVL3 |  |  | 321 | 0.148 | 0.2334 | Yes |
| 15 | PDE4DIP |  |  | 392 | 0.137 | 0.2370 | Yes |
| 16 | HIPK2 |  |  | 445 | 0.128 | 0.2420 | Yes |
| 17 | DPYSL3 |  |  | 464 | 0.127 | 0.2508 | Yes |
| 18 | SYF2 |  |  | 551 | 0.118 | 0.2509 | Yes |
| 19 | HDAC9 |  |  | 574 | 0.116 | 0.2583 | Yes |
| 20 | CREB1 |  |  | 577 | 0.116 | 0.2680 | Yes |
| 21 | CSNK1E |  |  | 580 | 0.116 | 0.2777 | Yes |
| 22 | NEDD4L |  |  | 615 | 0.113 | 0.2834 | Yes |
| 23 | RBP1 |  |  | 788 | 0.099 | 0.2720 | Yes |
| 24 | CDK6 |  |  | 807 | 0.097 | 0.2782 | Yes |
| 25 | QKI |  |  | 820 | 0.097 | 0.2851 | Yes |
| 26 | SOX2 |  |  | 863 | 0.093 | 0.2883 | Yes |
| 27 | TPGS2 |  |  | 913 | 0.090 | 0.2903 | Yes |
| 28 | HERC1 |  |  | 931 | 0.089 | 0.2960 | Yes |
| 29 | PFN2 |  |  | 942 | 0.089 | 0.3024 | Yes |
| 30 | UBE2N |  |  | 947 | 0.089 | 0.3096 | Yes |
| 31 | CBX3 |  |  | 966 | 0.087 | 0.3150 | Yes |
| 32 | NAV3 |  |  | 979 | 0.087 | 0.3210 | Yes |
| 33 | TOX3 |  |  | 999 | 0.086 | 0.3261 | Yes |
| 34 | MARCKS |  |  | 1081 | 0.081 | 0.3237 | Yes |
| 35 | SMARCE1 |  |  | 1194 | 0.075 | 0.3172 | Yes |
| 36 | RND3 |  |  | 1213 | 0.075 | 0.3216 | Yes |
| 37 | TIMM13 |  |  | 1220 | 0.075 | 0.3273 | Yes |
| 38 | TCF4 |  |  | 1234 | 0.074 | 0.3321 | Yes |
| 39 | EHBP1 |  |  | 1296 | 0.071 | 0.3312 | Yes |
| 40 | NIPSNAP1 |  |  | 1314 | 0.071 | 0.3353 | Yes |
| 41 | SLAIN1 |  |  | 1325 | 0.070 | 0.3401 | Yes |
| 42 | FXR1 |  |  | 1353 | 0.069 | 0.3429 | Yes |
| 43 | AP2M1 |  |  | 1422 | 0.067 | 0.3408 | No |
| 44 | PHF20L1 |  |  | 1887 | 0.051 | 0.2915 | No |
| 45 | ZBTB20 |  |  | 1892 | 0.051 | 0.2954 | No |
| 46 | CHD7 |  |  | 1953 | 0.049 | 0.2926 | No |
| 47 | ZFAND5 |  |  | 2017 | 0.047 | 0.2894 | No |
| 48 | NBEA |  |  | 2132 | 0.044 | 0.2800 | No |
| 49 | NCOA4 |  |  | 2207 | 0.041 | 0.2749 | No |
| 50 | CNOT7 |  |  | 2366 | 0.037 | 0.2598 | No |
| 51 | PSMG2 |  |  | 2443 | 0.035 | 0.2540 | No |
| 52 | DIMT1 |  |  | 2461 | 0.035 | 0.2550 | No |
| 53 | KLHL13 |  |  | 2530 | 0.033 | 0.2500 | No |
| 54 | NBPF1 |  |  | 2573 | 0.032 | 0.2478 | No |
| 55 | DCAF7 |  |  | 2575 | 0.032 | 0.2504 | No |
| 56 | ELF2 |  |  | 2638 | 0.030 | 0.2458 | No |
| 57 | SUCLA2 |  |  | 2844 | 0.026 | 0.2243 | No |
| 58 | NUP88 |  |  | 2873 | 0.025 | 0.2232 | No |
| 59 | RAB3IP |  |  | 2911 | 0.024 | 0.2209 | No |
| 60 | ATP6V1G1 |  |  | 2997 | 0.022 | 0.2130 | No |
| 61 | VEZF1 |  |  | 3022 | 0.022 | 0.2121 | No |
| 62 | DCLK2 |  |  | 3031 | 0.021 | 0.2130 | No |
| 63 | PLXNA2 |  |  | 3045 | 0.021 | 0.2132 | No |
| 64 | ABCA1 |  |  | 3161 | 0.019 | 0.2015 | No |
| 65 | ARMC1 |  |  | 3238 | 0.017 | 0.1942 | No |
| 66 | MTRNR2L1 |  |  | 3298 | 0.015 | 0.1887 | No |
| 67 | UBA3 |  |  | 3420 | 0.012 | 0.1758 | No |
| 68 | PPHLN1 |  |  | 3523 | 0.011 | 0.1649 | No |
| 69 | ARID4B |  |  | 3592 | 0.009 | 0.1579 | No |
| 70 | ALYREF |  |  | 3755 | 0.006 | 0.1396 | No |
| 71 | HIPK1 |  |  | 3800 | 0.005 | 0.1349 | No |
| 72 | PLXNA4 |  |  | 3824 | 0.004 | 0.1326 | No |
| 73 | GART |  |  | 3873 | 0.003 | 0.1274 | No |
| 74 | ZNF37A |  |  | 3902 | 0.002 | 0.1243 | No |
| 75 | GNPTAB |  |  | 3905 | 0.002 | 0.1243 | No |
| 76 | PCDH17 |  |  | 3912 | 0.002 | 0.1238 | No |
| 77 | SRGAP2B |  |  | 3959 | 0.001 | 0.1186 | No |
| 78 | ATRNL1 |  |  | 3976 | 0.001 | 0.1168 | No |
| 79 | RC3H2 |  |  | 4000 | 0.001 | 0.1142 | No |
| 80 | ANTXR1 |  |  | 4077 | -0.001 | 0.1055 | No |
| 81 | STAT2 |  |  | 4078 | -0.001 | 0.1056 | No |
| 82 | CD200 |  |  | 4124 | -0.002 | 0.1005 | No |
| 83 | HIGD1A |  |  | 4141 | -0.002 | 0.0988 | No |
| 84 | DMTF1 |  |  | 4218 | -0.003 | 0.0904 | No |
| 85 | RPRD1B |  |  | 4290 | -0.005 | 0.0826 | No |
| 86 | FAM107B |  |  | 4429 | -0.007 | 0.0672 | No |
| 87 | TIAM1 |  |  | 4472 | -0.008 | 0.0631 | No |
| 88 | NDUFA10 |  |  | 4640 | -0.011 | 0.0448 | No |
| 89 | LANCL1 |  |  | 4657 | -0.012 | 0.0439 | No |
| 90 | EIF1AX |  |  | 4719 | -0.013 | 0.0380 | No |
| 91 | ZEB2 |  |  | 4735 | -0.013 | 0.0374 | No |
| 92 | BRINP2 |  |  | 4802 | -0.014 | 0.0310 | No |
| 93 | KIAA1958 |  |  | 4819 | -0.015 | 0.0304 | No |
| 94 | GAD1 |  |  | 4858 | -0.016 | 0.0273 | No |
| 95 | ZC3H8 |  |  | 4958 | -0.017 | 0.0173 | No |
| 96 | SENP7 |  |  | 5076 | -0.020 | 0.0055 | No |
| 97 | MAP9 |  |  | 5139 | -0.021 | 0.0002 | No |
| 98 | NPEPPS |  |  | 5146 | -0.021 | 0.0013 | No |
| 99 | CHMP1B |  |  | 5220 | -0.022 | -0.0052 | No |
| 100 | SACS |  |  | 5232 | -0.023 | -0.0046 | No |
| 101 | MEIS1 |  |  | 5290 | -0.024 | -0.0091 | No |
| 102 | EPHA4 |  |  | 5330 | -0.025 | -0.0115 | No |
| 103 | LRRC40 |  |  | 5382 | -0.026 | -0.0151 | No |
| 104 | PLS3 |  |  | 5389 | -0.026 | -0.0136 | No |
| 105 | MIAT |  |  | 5393 | -0.026 | -0.0117 | No |
| 106 | ATF2 |  |  | 5459 | -0.028 | -0.0169 | No |
| 107 | LSAMP |  |  | 5577 | -0.030 | -0.0278 | No |
| 108 | UBE4B |  |  | 5698 | -0.032 | -0.0389 | No |
| 109 | ARMCX3 |  |  | 5743 | -0.033 | -0.0411 | No |
| 110 | B4GALT5 |  |  | 5833 | -0.035 | -0.0484 | No |
| 111 | TSPYL4 |  |  | 5991 | -0.038 | -0.0633 | No |
| 112 | RIC8B |  |  | 6034 | -0.039 | -0.0649 | No |
| 113 | CCDC88A |  |  | 6086 | -0.040 | -0.0674 | No |
| 114 | MFN1 |  |  | 6092 | -0.040 | -0.0645 | No |
| 115 | NCAM1 |  |  | 6342 | -0.046 | -0.0894 | No |
| 116 | UBR5 |  |  | 6426 | -0.048 | -0.0949 | No |
| 117 | MAPK10 |  |  | 6491 | -0.049 | -0.0981 | No |
| 118 | RAB18 |  |  | 6522 | -0.050 | -0.0972 | No |
| 119 | CCNE2 |  |  | 6916 | -0.062 | -0.1373 | No |
| 120 | PDE4D |  |  | 7047 | -0.066 | -0.1467 | No |
| 121 | ZNF618 |  |  | 7143 | -0.069 | -0.1518 | No |
| 122 | FNBP1L |  |  | 7173 | -0.070 | -0.1491 | No |
| 123 | SMARCD1 |  |  | 7216 | -0.071 | -0.1479 | No |
| 124 | ZNF704 |  |  | 7300 | -0.074 | -0.1511 | No |
| 125 | PDHB |  |  | 7466 | -0.080 | -0.1633 | No |
| 126 | SOX6 |  |  | 7616 | -0.087 | -0.1731 | No |
| 127 | FRA10AC1 |  |  | 7620 | -0.087 | -0.1660 | No |
| 128 | TMEM123 |  |  | 7786 | -0.095 | -0.1770 | No |
| 129 | VPS53 |  |  | 7847 | -0.098 | -0.1755 | No |
| 130 | ST8SIA5 |  |  | 7931 | -0.103 | -0.1763 | No |
| 131 | ANKRD17 |  |  | 8053 | -0.111 | -0.1809 | No |
| 132 | PSMA2 |  |  | 8122 | -0.116 | -0.1788 | No |
| 133 | GOLM1 |  |  | 8212 | -0.123 | -0.1786 | No |
| 134 | RNMT |  |  | 8277 | -0.129 | -0.1750 | No |
| 135 | TRAM1 |  |  | 8301 | -0.132 | -0.1663 | No |
| 136 | TMEM50A |  |  | 8329 | -0.136 | -0.1578 | No |
| 137 | CDCA7 |  |  | 8337 | -0.137 | -0.1469 | No |
| 138 | OCIAD1 |  |  | 8423 | -0.149 | -0.1440 | No |
| 139 | MIR181A1HG |  |  | 8461 | -0.154 | -0.1351 | No |
| 140 | COL4A3BP |  |  | 8516 | -0.165 | -0.1272 | No |
| 141 | DST |  |  | 8644 | -0.211 | -0.1238 | No |
| 142 | ZNF207 |  |  | 8655 | -0.215 | -0.1066 | No |
| 143 | CDH2 |  |  | 8695 | -0.237 | -0.0908 | No |
| 144 | DDOST |  |  | 8706 | -0.242 | -0.0712 | No |
| 145 | MEG3 |  |  | 8786 | -0.370 | -0.0487 | No |
| 146 | MALAT1 |  |  | 8796 | -0.581 | -0.0000 | No |
Table: GSEA details [plain text format]

  

Fig 2: RICHARDS\_NOWAKOWSKI\_SCIENCE\_2017\_NIN1\_UPREG: Random ES distribution      
 Gene set null distribution of ES for **RICHARDS\_NOWAKOWSKI\_SCIENCE\_2017\_NIN1\_UPREG**
